# Development and Psychometric Evaluation of the Adaptive Functions of Music Listening Scale

**DOI:** 10.3389/fpsyg.2018.00516

**Published:** 2018-04-12

**Authors:** Jenny M. Groarke, Michael J. Hogan

**Affiliations:** School of Psychology, National University of Ireland, Galway, Ireland

**Keywords:** music, functions of music listening, wellbeing, affect, regulation, psychometrics, scale development

## Abstract

Music listening may serve many adaptive functions in everyday life. However, studies examining the relationship between the functions of music listening (FML) and wellbeing outcomes have produced mixed findings. The purpose of this study is to develop a new measure to assess music listening functions that is psychometrically robust, and suitable for outcomes-based research on music listening and wellbeing. Scale items were developed based on a literature review and a prior qualitative enquiry. The items were reviewed by four content experts in music psychology and scale development. Scale structure was investigated by EFA and CFA in two large samples of participants (*N* = 1,191, 17–66 years, *M* = 22.04, *SD* = 6.23, 326 males). Tests of dimensionality revealed a 46-item scale with 11 factors for the Adaptive Functions of Music Listening (AFML) scale. Namely, *Stress Regulation, Anxiety Regulation, Anger Regulation, Loneliness Regulation, Rumination, Reminiscence, Strong Emotional Experiences, Awe and Appreciation, Cognitive Regulation, Identity*, and *Sleep* FML. The scale and its subscales possess good internal consistency and construct validity. In line with theory and research on gender differences in FML, scores on factors representing affect regulation FML were significantly higher among female respondents. Supporting the concurrent validity of the AFML scale, factors were positively correlated with an existing measure of the FML—the Music USE questionnaire. Further evidence of construct validity derives from positive associations between affect regulation factor scores and level of reappraisal, and lack of association with suppression, as measured by the Emotion Regulation Questionnaire. Consistent with the view that adaptive FML are positively related to wellbeing, a number of factors, affect regulation factors in particular, were significantly positively correlated with subjective, psychological, and social wellbeing measures across two cross-sectional studies.

## Introduction

Listening to music is a common behavior engaged in by most people from childhood through to adulthood and into advanced old age (Laukka, 2007; Juslin et al., 2008). Music listening may serve many adaptive functions for individuals across their lifespan. Questionnaire and survey studies have been generative in providing lists of common functions of music listening (FML). A number of these studies have also uncovered a latent factor structure of the FML using cluster or factor analysis—but further scale development and validation efforts to create instruments to measure the FML have been slower to emerge. Previous investigations have found that the FML are broadly affective, cognitive, and social (Hargreaves and North, 1999). In a review of twenty eight empirical studies, Schäfer et al. (2013) identified 129 FML which they synthesized into three high-level functions: arousal and mood regulation, self-awareness, and social relatedness. This synthesis was based on the item-level analysis of existing FML scales and taxonomies. Qualitative studies have highlighted a wider range of other potentially adaptive FML including goal-attainment, cognitive control, transcendence, absorption, and flow, amongst others (Hays and Minichiello, 2005; Lamont, 2011; Herbert, 2012; Schäfer et al., 2014; Groarke and Hogan, 2016). While a number of FML scales have been developed to measure specific functions of music listening, such as mood regulation (Saarikallio, 2008), or specific musical experiences like absorption (Sandstrom and Russo, 2013), the current study sought to validate a general measure of the FML. Table 1 provides a summary of the scale structure and psychometric properties of five existing global measures of the FML identified in the literature. A review of Table 1 suggests there is a need for a psychometric measure of the functions of music listening that incorporates a larger set of FML in a single multi-factorial scale.

**Table 1:** Development and scale structure of general measures of the functions of music listening.

**Name of measure**	**Author (year)**	**Item development**	**Item wording**	**Response format**	**Factor analysis**	**Scale structure**
Uses of music inventory	Chamorro-Premuzic and Furnham, 2007	Unknown number of items representing 3 FML constructs Literature review Focus groups, interviews with students to identify constructs	Effect of music	Agreement	Sample 1 PCA Varimax rotation	1 Emotional uses of music (5 items) 2. Rational/cognitive uses of music (5 items) 3. Background use of music (5 items) 15 items
Music USE questionnaire: music engagement styles	Chin and Rickard, 2012	124 items representing 4 FML constructs Literature review Focus groups to evaluate face validity of items (music psychology scholars and students)	Effect of music	Applicability to me	Sample 1 PCA Varimax rotation Sample 2 EFA (Maximum Likelihood Extraction) Varimax rotation	1. Cognitive and emotional regulation (7 items) 2. Engaged production (9 items) 3. Social connection (3 items) 4. Dance (2 items) latha5. Physical exercise (3 items) 24 items
Motives for listening to music questionnaire	Kuntsche et al., 2016	12 items representing 4 FML constructs Adapted from the motivational model of alcohol use (Cox and Klinger, 1988)	Outcome sought	Frequency of use	Sample 1 CFA	1. Enhancement (3 items) 2. Coping (3 items) 3. Social (3 items) 4. Conformity (3 items) 12 items
The music experience questionnaire	Werner et al., 2006	141 items representing unknown number of FML constructs Literature review Discussion to evaluate face validity of items (musicians, non-musicians, and music psychology students)	Effect of music	Accuracy of statements describing their reactions to music	Sample 1 Item reduction by item to scale analyses Sample 2 EFA (principal factors analysis) Varimax rotation	1. Affective reactions (7 items) 2. Positive psychotropic effects (16 items) 3. Reactive musical behavior (9 items) 4. Commitment to music (7 items) 5. Innovative musical aptitude (7 items) 6. Social uplift (4 items) 53 items
The Barcelona music reward questionnaire	Mas-Herrero et al., 2013	112 items representing 6 FML constructs Literature review Focus groups to evaluate face validity of items (musicians and non-musicians)	Effect of music	Agreement	Sample 1 EFA of polychoric correlation matrix Oblique rotation Sample 2 CFA	1. Emotional evocation (4 items) 2. Sensory-motor (4 items) 3. Mood regulation (4 items) 4. Musical seeking (4 items) 5. Social reward (4 items) 20 items

A survey of the literature suggests that music listening may produce effects that relate to a number of adaptive outcomes, such as enhanced wellbeing. For instance, music may enhance wellbeing by inducing positive affective experiences (Juslin et al., 2008), regulating negative affective experiences (Knight and Rickard, 2001; Sandstrom and Russo, 2010; Radstaak et al., 2014), enhancing social functioning (Hays and Minichiello, 2005; North and Hargreaves, 2006; Miranda and Claes, 2009), and giving rise to experiences such as transcendence, peak experience, and flow (Gabrielsson, 2010; Lamont, 2011; Harrison and Loui, 2014).

At the same time, there have been relatively few direct investigations of the relationship between self-reported functions of music listening and specific wellbeing outcomes (Laukka, 2007; Thoma et al., 2012; Chin and Rickard, 2014a,b; Papinczak et al., 2015; Saarikallio et al., 2015; Randall and Rickard, 2017). The limited number of studies is perhaps due to the lack of suitable measures. Some of these studies have used well-developed psychometric measures of FML (Chin and Rickard, 2014a,b; Saarikallio et al., 2015), whereas other studies have used a limited number of researcher-selected lists of FML without carrying out further scale development or validation. This suggests that a new integrative measure of music listening functions that is psychometrically sound may be a useful addition to the emerging field of music, health, and wellbeing. Contrary to expectations, some studies examining relationships between FML and wellbeing highlight some negative effects of music listening on measures of wellbeing. For example, among university students, listening to music “to reduce loneliness” predicted lower quality of life (Thoma et al., 2012). Similarly, Randall and Rickard (2017) found that more frequent listening to music for emotional reasons (e.g., “to cope,” “to forget”) was related to higher depression and anxiety.

Integrating research on the functions of music listening is difficult due to the varied conceptual definitions of constructs used across studies (i.e., outcomes, effects, responses, goals, reasons, rewards, reactions, experiences, uses, motives, and functions of music listening). For example, there is an important distinction to be made between the outcome or effect a listener is pursuing (i.e., the goal, motive, reason) and the outcome they actually experience listening to music (i.e., the outcome, effect, response, reaction). Relationships with various outcomes, including wellbeing, would be expected to differ based on this distinction. In the current project, in an effort to support greater synthesis, a long-standing theory of learning and behavior, Social Cognitive Theory (Bandura, 1989, 2001) underpins the development of items for the *The Adaptive Functions of Music Listening Scale* (AFML scale).

Social Cognitive Theory (SCT) would predict that music listening behavior is guided by listeners' beliefs about the effects of music (i.e., self-efficacy) and their expectations of achieving particular outcomes by listening to music (i.e., outcome expectations). The listener's beliefs and expectations about the effects and outcomes of music are acquired through experience and vicarious learning. With time, these effects and outcomes provide incentives that become goals guiding the behavior of music listening. When such goal-directed behavior is effective, outcomes of musical experiences become functions that music listening serves in an individual's life.

The application of SCT as an approach to scale item development undertaken in the current study is also in line with recent theoretical developments in music psychology, where goals and effects are increasingly being seen as important facets of the music listening experience (van Goethem and Sloboda, 2011; Baltazar and Saarikallio, 2016; Schäfer, 2016). The AFML scale asks participants to rate their level of agreement with statements about the efficacy of achieving a range of potential adaptive outcomes when listening to music. This allows researchers to test the hypothesis that endorsing an adaptive function of music listening is indeed associated with an adaptive outcome, such as the enhancement or maintenance of wellbeing.

This approach relies on the retrospective self-reports of listeners, which can be limited by recall bias and influenced by commonly held positive beliefs about the efficacy of music for different functions. An alternative approach would be to use Experience Sampling Methodologies (ESM) to measure an individual's goal or function of music listening prior to an episode of music listening, and then measure the efficacy of music listening for that function in that individual episode, as well as over the course of all music listening episodes sampled. Clearly this is a powerful methodological approach. Yet, there are some drawbacks that limit the application of ESM in all research contexts. For instance, the pagers and palmtop computers that have been used to randomly sample episodes of everyday life are prohibitively expensive and ESM studies of music using them have had relatively small sample sizes (Sloboda et al., 2001; Juslin et al., 2008). Other studies have used text messages to prompt participants to complete questionnaires at random intervals throughout the day (North et al., 2004; Greasley and Lamont, 2011). While this approach is cost-effective, the potential for response forms to be back-filled or forward-filled reduces the validity of the data. A new tool for conducting ESM studies on music listening is now available: The MuPsych application (Randall and Rickard, 2013). The MuPsych app collects data in real-time using pop-up questionnaires presented on the participant's smartphone allowing for ecologically valid data collection in naturalistic settings. In general, participant burden is high in ESM studies as participants are requested to carry new devices or questionnaires at all times for the duration of the study. There is also some concern that participant burden would be higher for older people who may struggle to adopt new devices and technologies (i.e., pagers, palmtops, smartphones and portable music players) (Zhou et al., 2014). Experience sampling is certainly a robust method of studying adaptive FML in context and over time. However, it may be less suitable for collecting data with older cohorts, or for studies requiring large sample sizes, such as scale development work.

The current study followed guidelines for scale development advocated by DeVellis (2012). This involves following a series of steps in the scale development process, specifically, conducting a thorough review of the literature surrounding the constructs to be measured, generating an exhaustive item pool, selecting an appropriate response format, item evaluation by experts, application of both exploratory and confirmatory factor analysis in separate scale development samples, alpha reliability assessment, and optimizing scale length.

Qualitative work has been identified as the ideal starting point for scale construction, particularly for item development and identifying valid domains to include in item pools (O'Brien, 1993; Rowan and Wulff, 2007; DeVellis, 2012). The conceptual framework informing scale development in the current study arose out of focus group style sessions with music listeners (Groarke and Hogan, 2016) and a review of existing FML research, with a particular focus on research linking music listening and wellbeing. The initial conceptual model informing the development of scale items is presented in **Figure 2**.

A summary overview of the adaptive functions of music listening identified by literature review and qualitative enquiry, and their relevance for wellbeing outcomes and construct validation are described below. These include affective, social, eudaimonic, and cognitive functions of music listening.

Affective functions, such as mood and emotion regulation, are the most commonly reported reasons for music listening (Tarrant et al., 2000; Schäfer et al., 2013). Wellbeing research and theory highlights important distinctions between positive and negative affective experiences, between affective experience and affective regulation, and distinguishes between subjective, psychological, and social dimensions of wellbeing. Scale items on existing FML measures have not been developed in line with these distinctions limiting our ability to make theoretically-derived hypotheses regarding potential relationships between these discrete FML and wellbeing outcomes.

Affective experiences can be distinguished by valence (positive and negative) and arousal (high arousal and low arousal) dimensions (Russell, 1980; Watson and Tellegen, 1985), and many studies have demonstrated that music listening can induce a range of affective experiences that vary along these dimensions (Juslin et al., 2008). However, existing FML measures have not always distinguished affective experiences in music listening along these dimensions. For example, The Barcelona Music Reward Questionnaire; (Mas-Herrero et al., 2013) includes an affective factor measuring *emotional evocation*, which includes general items such as “I get emotional listening to certain pieces of music.” The Uses of Music Inventory (Chamorro-Premuzic and Furnham, 2007) includes a factor to measure *emotional use of music*—participants rate their agreement with statements such as “Listening to music really affects my mood.” Such items become problematic when trying to predict relationships between FML factor scores and wellbeing, as they do not distinguish the valence of the emotion referred to, and research shows that positive and negative affective experiences can independently predict wellbeing outcomes (Diener et al., 2004). Therefore, it is important to develop a measure of the FML that distinguishes a range of affective experiences that can be stimulated by music. As shown in **Figure 2**, these include positive and negative affect, strong and mixed emotional experiences and reminiscence (Sloboda, 1999; Gabrielsson, 2010; Schäfer et al., 2014). It is expected that scores on affective experience factors will associate positively with both higher positive affect (PA) and negative affect (NA).

Affect regulation strategies influence the frequency and intensity of ongoing affective experience (Folkman and Lazarus, 1988). As noted above, positive and negative affective experiences induced by music listening may relate differentially to wellbeing, whereas, the successful regulation of NA in music listening should relate to increased wellbeing (Larsen, 2009). However, with the exception of The Barcelona Music Reward Questionnaire (Mas-Herrero et al., 2013) FML scales have not distinguished between affective experience and affective regulation FML.

Every FML measure reviewed in Table 1 has extracted at least one factor measuring affect regulation. However, a drawback of these global FML scales is that affect regulation factors do not always enable simultaneous measurement of the strategies by which music can regulate negative affective experiences. For example, items measuring *cognitive and emotional regulation* on the MUSE questionnaire include “I often listen to music when I'm feeling down” and “Specific types of music makes me feel better” (Chin and Rickard, 2012). At the same time, music psychology research has pointed to a substantial range of different strategies listeners use to regulate affective experience, such as relaxation, distraction/diversion, venting/discharge, mental work/rational thinking, solace/comfort, and rumination (Saarikallio and Erkkila, 2007; Saarikallio, 2011; van Goethem and Sloboda, 2011). Some of these strategies correspond with the general affect regulation literature, which, in the context of mainstream health psychology points to various behavioral and cognitive, emotion-focused and problem-focused regulation strategies (Lazarus and Folkman, 1984; Carver et al., 1989; Gross, 1998; Larsen, 2000). Item development for the AFML scale builds upon this theoretical and empirical research and proposes a diverse and comprehensive range of regulation strategies by which listening to music may regulate NA. Namely, distraction, venting, reappraisal, emotional support, emotional approach, generating positive emotions, escape, and rumination (see **Figure 2**). The wider literature also suggests regulation strategies may vary in terms of their relationship with wellbeing outcomes (Gross and John, 2003; Aldao et al., 2010). Therefore, including diverse affect regulation FML in scales will be useful for research questions regarding the efficacy of different musical affect regulation strategies for wellbeing enhancement.

Given the importance of affect regulation for wellbeing, it is expected that affect regulation FML will be associated with higher subjective wellbeing (SWB), specifically, lower negative affect, higher positive affect and higher reported life satisfaction.

Most existing measures of the FML have extracted a factor relating to the social functions of music (see Table 1). Factors measuring increased connection and bonding between listeners over shared musical tastes and group listening experiences have been determined (Chin and Rickard, 2012; Mas-Herrero et al., 2013). Other measures have focused on the value of music in social situations, such as increased atmosphere and celebration (Kuntsche et al., 2016). Therefore, music listening may have an important function in the development and maintenance of positive relationships with others. At the same time, using existing measures, the relationship between the social functions of music and wellbeing outcomes have not been firmly established. For instance, in one study, the factor measuring *social connection* on the MUSE questionnaire was not associated with enhanced subjective, psychological or social wellbeing; rather it was significantly associated with increased use of the emotion regulation strategy of suppression, which predicted lower levels of wellbeing (Chin and Rickard, 2014a). A survey study of younger adults by Papinczak et al. (2015) found that higher social wellbeing was predicted by the total effect of four FML (i.e., relationship building, immersing in emotions, modifying emotions, modifying cognitions). Theoretically, social FML should be related to greater psychological wellbeing (Ryff and Keyes, 1995) and social wellbeing (Keyes, 1998), however, empirical support is lacking.

In relation to the social function of identity, although further scale development work was not undertaken, identity FML have been extracted using Principal Components Analysis (PCA) in survey studies with adolescent (Lonsdale and North, 2011) and older samples (Laukka, 2007). In line with theory and research it is predicted that if identity FML are uncovered in factor analysis in the current study, they may relate to greater SWB through increased positive affect (Kahn et al., 1985), and higher psychological wellbeing through increased self-acceptance, psychological growth, and meaning (Ryff, 1989; Pennebaker and Seagal, 1999; Haslam et al., 2009).

Previous investigations have uncovered a number of FML that could be described as eudaimonic functions. These include music-induced peak experiences (Maslow, 1999; Gabrielsson, 2010), transcendence (Hays and Minichiello, 2008; Schäfer et al., 2014), and engagement or flow (Lamont, 2011). Participants engaged in focus groups voted that a number of these FML (i.e., transcendence and meaning) were beneficial for wellbeing enhancement (Groarke and Hogan, 2016). Outside of musical contexts, such eudaimonic experiences, particularly transcendence, have been associated with increased happiness and life satisfaction, and greater meaning in life (Gillham et al., 2011). Empirical studies of FML have tended not to include items to measure these eudaimonic functions, thus it remains to be seen whether eudaimonic experiences in music listening also relate positively to subjective, psychological, and social wellbeing outcomes.

Factors relating to listening to music for its cognitive effects are a focus of existing measures of the FML (Chamorro-Premuzic and Furnham, 2007; Chin and Rickard, 2012). Cognitive functions include music analysis (Chamorro-Premuzic and Furnham, 2007), and it is possible that the pleasure derived from the analysis of music may relate to enhanced SWB. A more reflective style of music listening may also provide a sense of awe and appreciation, stimulating self-reflection and insight (Cupchik, 1995; Groarke and Hogan, 2016), which may theoretically relate to higher psychological wellbeing (Ryff and Singer, 2008).

The use of music to regulate cognitive states like curiosity and creativity, as well as focus, attention, and motivation have been noted in surveys (North et al., 2000; Tarrant et al., 2000) and measures of the FML (Chamorro-Premuzic and Furnham, 2007; Chin and Rickard, 2012). These effects of music may support listeners in the achievement of everyday goals that depend on cognitive engagement and proficiency (DeNora, 2000; Groarke and Hogan, 2016). Goal-attainment and achievement are central to models of wellbeing (Ryan and Deci, 2000; Seligman, 2012), and have been related to emotional wellbeing in empirical research (Schultheiss et al., 2008). Therefore the pursuit of such cognitive goals by music listening may also relate to increased wellbeing. This view is consistent with some available research. For example, the *cognitive and emotion regulation* factor on the MUSE questionnaire was associated with higher subjective, psychological and social wellbeing (Chin and Rickard, 2014a,b). These effects were fully mediated by increased use of the affect regulation strategy reappraisal. Qualitative research has also proposed that cognitive regulation in music may support affect regulation goals (DeNora, 1999; Papinczak et al., 2015). Thus, it is hypothesized that cognitive FML may be associated with higher self-reported wellbeing in the current study.

The construct validity of a measure is assessed by forming theoretically-based hypotheses regarding potential relationships with other measures (Carmines and Zeller, 1979). In this study construct validity will be assessed using convergent validity, that is, how closely scores on the measure under development converge with scores on other measures of related constructs (Furr and Bacharach, 2008). In line with theory and research summarized above, significant associations should be observed between FML constructs as measured by the AFML scale and measures of affect, affect regulation, and wellbeing. A measure's construct validity can also be established using concurrent validity, that is, by demonstrating even greater convergence with scores on other measures of the same construct. In the current study the well-validated measure of affective, social, and cognitive FML—The Music USE Questionnaire (MUSE) (Chin and Rickard, 2012) will be employed to test the concurrent validity of the AFML scale.

## Materials and methods

### Design

The current scale development study involved two phases: Firstly, the initial scale development phase involved item generation, assessing the dimensionality of the measure using EFA, reducing the initial pool of questionnaire items, and examining the reliability and construct validity of the AFML scale. The second phase involved confirming the factor structure derived from EFA in a separate sample of participants (recruited 9 months later). In the first phase construct validity was assessed by testing hypothesized relationships between AFML factors and subjective wellbeing outcomes. In the second confirmatory phase additional relationships between FML factors and psychological and social wellbeing outcomes were examined, in addition to relationships with a general measure of emotion regulation, and an existing general measure of music listening functions.

### Initial scale development

#### Item generation

Two-hundred and forty items were generated on the basis of an extensive literature review and four focus group sessions, with two younger adult groups (*N* = 25, *M* = 22.49 years, *SD* = 2.25) and two older adult groups (*N* = 19, *M* = 65.86, *SD* = 4.46). At least five items and one reverse scored item were generated for 38 hypothesized functions of music listening derived from these sessions.

#### Content validity

Four content experts on scale development (3 in music psychology, 1 in psychometrics) responding to an online questionnaire rated these 240 items for their clarity, relevance, and comprehensiveness. The experts made a number of suggestions to increase the clarity and meaningfulness of items, removal of redundant items, and restructuring of the affect regulation subscales to allow for more differentiated responding. Overall, 164 items were rated by experts as relevant and were retained for EFA.

#### Pilot testing

The 164-item AFML measure was administered to 9 lay experts, or potential participants (4 male, 18–30 years, *M* = 21.55, *SD* = 4.80) for pilot testing. All 164 items were rated as very or quite clear. Although participants rated the items highly, they also reported that the questionnaire was long and repetitive in certain respects. However, all items were retained for factor analysis in order to identify the items of highest psychometric quality for inclusion in the final AFML scale. DeVellis (2012) emphasizes the need for a large pool of items, and multiple indicators for each hypothesized construct at the development stage. Therefore, multiple indicators (including 1 reverse scored item) representing each of the remaining 33 hypothesized FML were administered to a development sample for item reduction and EFA.

### Further scale development

#### Procedure

Potential participants were invited to take part in an online survey of why they listen to music via online advertisements, university email campaigns, and national media. All participants provided informed consent, and completed the questionnaire packet online using Survey Gizmo.

#### Participants

##### Development sample

In the development phase, 1,396 participants initially consented to take part. 673 participants (452 Females) completed all items in the online questionnaire packet (48% completion rate).

##### Confirmatory sample

Of 1,267 prospective participants who consented to take part in the online questionnaire at the confirmatory stage, 47% completed it (*N* = 597 participants, 413 Females).

Seventy three percent of the development sample and 55% of the confirmatory sample were undergraduate psychology students receiving research participation credit. One instructed item was included to identify insufficient effort responding (i.e., please select the “neutral” response option). Thirty seven participants were removed from the EFA analysis for failing to select the correct response to the instructed item. The remaining 637 respondents included in EFA analyses were mostly female (68%) university students (98%). In the confirmatory factor analysis, thirty two participants were removed for insufficient effort responding, and the remaining 554 participants were mostly female (69%) university students (87%). The final sample included in analyses in both phases of the study includes only those participants who completed all items in the online questionnaire, and who selected the correct response to the instructed item.

#### Materials

##### Development sample

In addition to demographic questions (i.e., age, gender, educational, and occupational status), participants completed the following measures:

*The adaptive functions of music listening scale—(AFML scale)*. Participants rate their level of agreement with 164 items representing outcome expectations of a range of music listening functions using a 5-point Likert scale ranging from 1 (strongly disagree) to 5 (strongly agree). The AFML scale has a Flesch-Kincaid Grade level of 5 and a Flesch Reading Ease score of 83.8 placing it in the “easy” range (Flesch, 1948; Kincaid et al., 1975).

*Music engagement intensity subscale of the Music USE questionnaire (MUSE) (Chin and Rickard, 2012)*. This 8-item measure provides 3 indices of music engagement. Scores range from 1 to 25 on the Index of Music Listening (IML), with higher scores indicating more intense music listening. The Index of Music Training (IMT) assesses an individual's music education, higher scores indicate more musical training. The Index of Music Instrument Playing (IMIP) provides a total score based on respondents' years of instrument playing, hours of practice per day and regularity of practice. Higher scores represent greater engagement with instrument playing.

##### Subjective wellbeing

*Positive and negative affect schedule (PANAS) (Watson et al., 1988)*. The PANAS consists of 20 adjectives: 10 describe positive emotions and 10 describe negative emotions. Participants indicate the extent to which they have experienced these emotions in the previous week, using a Likert scale ranging from “very slightly or not at all” (1) to “extremely” (5). Two sub-scale scores are derived, with higher scores indicating greater positive and negative affect, respectively.

*The Satisfaction with Life Scale (SWLS) (Diener et al., 1985)*. Participants indicate their level of agreement with 5 life satisfaction statements, using a 7-point scale that ranges from 7 (strongly agree) to 1 (strongly disagree).

*Confirmatory sample*. In addition to the measures completed by participants in the development phase, the participants in the confirmatory sample completed a number of additional measures to further assess construct validity:

*Adaptive functions of music listening scale*. A shortened version of the AFML scale with 48 items was administered (based on EFA results). Responses to statements of potential outcomes of music listening were made on a 5-point Likert-scale ranging from 1 (Strongly Disagree) to 5 (Strongly Agree). Subscale scores are calculated by averaging scores across items.

*Mental health continuum- short form (MHC-SF) (Lamers et al., 2010)*. The MHC-SF is a 14-item measure of positive psychological functioning. It assesses three dimensions of wellbeing: Emotional, Psychological and Social wellbeing. Respondents rate the frequency of various feelings of wellbeing on a 6-point Likert scale (never - everyday) and scores are then summed. The emotional wellbeing dimension is an alternative 3-item measure of SWB, thus only Psychological and Social wellbeing scores on the MHC-SF will be presented.

*The Music USE Questionnaire (MUSE) (Chin and Rickard, 2012)*. The Music Engagement Styles (MES) subscales measure five styles of music listening. These styles are (i) cognitive and emotional regulation (7items), (ii) engaged production (9 items), (iii) social connection (3 items), (iv) physical exercise (3 items), and (v) dance (2 items). Respondents rate their agreement with a series of statements (e.g., “I often listen to music when I'm feeling down”) on a 6-point Likert scale from “0” (not at all/not applicable to me) to “5” (strongly agree). Scores are summed.

*Emotion Regulation Questionnaire (ERQ) (Gross and John, 2003)*. The ERQ is a 10 item measure composed of two subscales: Reappraisal and Suppression. Reappraisal is the cognitive restructuring of thoughts to increase positive and reduce negative emotions, whereas suppression involves inhibiting the expression of emotion. Scores, ranging from 1 (strongly disagree) to 7 (strongly agree), are summed for each subscale.

Descriptive statistics and Cronbach's alpha for all of the measures included in this study with both samples of participants are presented in Table 2.

**Table 2:** Participant characteristics and descriptive statistics for all measures included in two phases of data collection with the development sample and a separate confirmatory sample.

		**Development sample (*N* = 637)**			**Confirmatory sample (*N* = 554)**		
**Study variables**	**Test range**	**Sample range**	**M *(SD)***	**α**	**Sample range**	**M *(SD)***	**α**
Age		17–66	22.19 (6.25)		17–66	21.88 (6.20)	
Index music listening	1–25	1–25	12.17 (5.75)		2–25	11.34 (5.76)	
Index musical instrument playing		0–575	20.23 (42.37)		0–420	12.66 (28.72)	
Index musical training	0–11	2–10	6.75 (1.51)		4–11	8.39 (1.59)	
**SUBJECTIVE WELLBEING**							
Positive affect	10–50	10–50	32.22 (7.84)	0.88	10–44	28.83 (7.17)	0.89
Negative affect	10–50	10–48	22.80 (8.18)	0.87	10–47	21.52 (7.75)	0.87
Satisfaction with life	5–35	5–35	22.59 (7.20)	0.89	5–35	22.51 (6.50)	0.85
**MENTAL HEALTH CONTINUUM**							
Psychological wellbeing	0–35				0–30	20.14 (5.35)	0.85
Social wellbeing	0–25				0–25	14.60 (4.61)	0.81
**MUSE**							
MUSE-total scale score	0–120				0–120	74.33 (20.74)	0.91
Cognitive and emotional regulation	0–35				0–35	27.89 (4.72)	0.78
Engaged production	0–45				0–45	18.49 (14.85)	0.95
Social connection	0–15				0–15	10.94 (2.90)	0.86
Physical exercise	0–15				0–15	11.74 (3.16)	0.78
Dance	0–10				0–10	5.30 (3.33)	0.69^r^
**ERQ**							
Reappraisal	6–42				6–35	24.72 (5.03)	0.83
Suppression	4–28				4–28	15.10 (5.12)	0.64

*MUSE, Music USE Questionnaire; ERQ, Emotion Regulation Questionnaire; M, Mean; SD, Standard Deviation; α, Cronbach's alpha coefficient*;

r *, Pearson's r (two item scale)*.

## Results

### Dimensionality

#### Exploratory factor analysis—development sample

An Exploratory Factory Analysis (Principal Axis Factoring) was carried out using SPSS version 22. Factors representing music listening functions were expected to correlate therefore oblique rotation using Direct Oblimin with Kaiser Normalization was deemed appropriate. The Kaiser Meyer-Olkin measure (KMO = 0.95), and a significant Bartlett's test of sphericity [$X_{\left(1035\right)}^{2}$ = 17,454.06, *p* < 0.001] indicated suitability of the dataset for factor analysis.

Factor retention decisions were made on the basis of Horn parallel analysis 1965, the Kaiser criterion (Eigenvalues greater than 1) (Kaiser, 1960), visual inspection of the Scree plot (Cattell, 1966), the proportion of variance explained (Beavers et al., 2013), as well as conceptual considerations.

Consistent with standard practice, items were retained if they had loadings in excess of 0.40, no cross-loadings above 0.32, and item communalities over 0.40 (Worthington and Whittaker, 2006). Each factor was assessed for the presence of redundant items, and within factors inter-item correlations were between 0.30 and 0.90. Item-total correlations were required to be above 0.30 to allow averaging of factor scores without applying item weights (Field, 2009).

The related but distinct processes of factor analysis and item deletion should be carried out iteratively (Worthington and Whittaker, 2006). This process involves removing items from the analysis, one at a time, repeating the EFA and comparing the solutions using multiple criterion methods (i.e., parallel analysis, the Kaiser rule, percentage of variance explained, and the scree plot) (Costello and Osborne, 2005; Schönrock-Adema et al., 2009).

Applying Principal Axis Factoring (PAF) to the 164-item dataset, 32 factors were extracted accounting for 60.40% of the variance. One hundred and sixteen items were deleted iteratively for failing to meet item retention criteria. Using syntax provided by O'Connor (2000) parallel analysis of this 48-item dataset recommended a 13-factor solution (63.88% variance explained), and the Kaiser criterion suggested 8 factors be retained accounting for 56.72% of the variance. Forcing 8 factors led to a solution that was not sufficiently conceptually differentiated. The 13-factor solution suggested by parallel analysis contained 4 factors that, on theoretical grounds and by reference to the existing literature, were better represented as 2 factors. Ultimately, an 11-factor solution was deemed most parsimonious and comprehensive and accounted for 61.78% of the variance. This decision was guided primarily by parallel analysis in conjunction with theoretical considerations, while aiming to maximize the proportion of variance explained. In addition, all 48 items and 11 factors possessed good psychometric properties set forth by item and factor retention criteria above, and are reported in Table 3.

**Table 3:** Results of EFA and psychometric properties of 11 factors of the AFML scale.

**AFML factors**	**% variance**	**Loading (range)**	**Loading (M)**	**α**	**Eigen value**	**M *(SD)***
1. Stress regulation (4 items)	27	0.52–0.62	0.59	0.85	12.87	4.08 (0.71)
2. Strong emotional experiences (6 items)	11	0.58–0.79	0.69	0.90	5.29	3.98 (0.71)
3. Rumination (5 items)	5	0.56–0.78	0.67	0.82	2.24	3.13 (0.81)
4. Sleep (2 items)	4	0.89–0.92	0.91	0.84^r^	1.90	3.08 (1.22)
5. Reminiscence (4 items)	3	0.60–0.79	0.71	0.82	1.52	4.20 (0.64)
6. Anger regulation (7 items)	3	0.44–0.79	0.66	0.91	1.27	3.64 (0.85)
7. Anxiety regulation (7 items)	2	0.54–0.76	0.65	0.91	1.16	3.93 (0.72)
8. Awe and admiration (3 items)	2	0.63–0.85	0.76	0.83	1.04	4.09 (0.73)
9. Loneliness regulation (3 items)	2	0.74–0.84	0.78	0.83	0.92	3.88 (0.77)
10. Cognitive regulation (2 items)	2	0.84–0.87	0.86	0.75^r^	0.86	3.11 (1.12)
11. Identity (5 items)	1	0.50–0.90	0.63	0.86	0.66	3.73 (0.84)
AFML-total scale (48 items)	62			0.94		

*N = 637; M, Mean; SD, Standard Deviation; α, Cronbach's alpha coefficient*;

r *, Pearson's r (two item scale)*.

#### Confirmatory factor analysis—confirmatory sample

In a separate confirmatory sample of participants, CFA was conducted using Structural Equation Modeling (SEM) in Amos version 23 (Arbuckle, 2014). Model fit was assessed using a number of indices. Firstly, a non-significant chi square test is indicative of a well-fitting model. The normed chi-square (*Q*) is the chi square index divided by the degrees of freedom: acceptable criteria vary from under 2 (Ullman, 2001) to less than 5 (Schumacker and Lomax, 2004). The comparative fit index (CFI) was also used: values at or greater than 0.90 and 0.95 reflect acceptable and excellent fit to the data, respectively (Kenny and McCoach, 2003). Finally, we used the root mean square error of approximation (RMSEA), with values between 0.05 and 0.09 indicating adequate model fit and values below 0.05 indicating a very good fit (Hu and Bentler, 1999). Modification indices available in CFA can be used to identify misspecification in the model. Decisions regarding modifications were based on theoretical and psychometric considerations of item and scale content. We would not allow error residuals to covary, however we would eliminate items if they had low factor loadings (i.e., standardized regression coefficients) (<0.60), or if modification indices suggested they had significant loadings (>0.30) with unintended latent factors (Byrne, 2010).

The 11-factor solution identified using EFA was tested with CFA with a separate sample of participants. The initial model specified was the 48 scale items loading onto their respective factors. This initial measurement model was an acceptable fit: $X_{\left(1025\right)}^{2}$ = 2,178.92, *p* < 0.001, *Q* = 2.13, CFI = 0.93, RMSEA = 0.045 (90% CI, 0.042–0.048). One item was removed from the *Rumination* factor, and one from the *Identity* factor for failing to load above 0.60. These two items were also deemed less conceptually related to the other items. This led to improvement in model fit, and this final model represented a very good fit of the data: $X_{\left(946\right)}^{2}$ = 1,879.33, *p* < 0.001, *Q* = 1.99, CFI = 0.94, RMSEA = 0.042 (90% CI, 0.039–0.045). The final 46 items of the AFML scale and their beta weights (β), that is their factor loadings, as well as, the proportion of variance in the latent construct explained by that item (*r*^2^) in the confirmatory sample are reported in Table 4.

**Table 4:** The adaptive functions of music listening scale, internal consistency and descriptive statistics of 11 subscales, and psychometric properties of 46 final scale items.

**The adaptive function of music listening scale**	**β**	***r*^2^**	**α**	**M *(SD)***
**Stress regulation**			0.85	3.97 (0.64)
1. Listening to music distracts me from stress	0.74	0.54		
2. When I feel stressed listening to music helps to take my mind off it	0.78	0.60		
3. I can escape from stressful situations by listening to music	0.77	0.59		
4. When I feel stressed I get comfort from listening to music	0.75	0.56		
**Strong emotional experiences**			0.90	3.85 (0.73)
1. When listening to music I feel intense emotions	0.83	0.68		
2. When listening to music I feel a range of emotions	0.78	0.61		
3. When listening to music I feel emotions deeply	0.81	0.66		
4. When listening to music I feel a variety of emotions simultaneously	0.71	0.50		
5. When listening to music I feel a mixture of many different emotions	0.75	0.56		
6. I feel strong emotions when listening to music	0.81	0.67		
**Rumination**			0.80	2.91 (0.80)
1. When I feel sad/depressed listening to music makes me dwell upon those feelings	0.77	0.59		
2. When I feel sad/depressed listening to music leads me to focus on those feelings	0.77	0.59		
3. When I feel anxious listening to music makes me dwell upon those feelings	0.67	0.45		
4. When I feel anxious listening to music leads me to focus on those feelings	0.62	0.39		
**Sleep**			0.87^r^	3.00 (1.23)
1. Listening to music in bed helps me fall asleep	0.88	0.78		
2. I listen to music in bed because it helps me get to sleep	0.99	0.98		
**Reminiscence**			0.88	4.10 (0.73)
1. Listening to music does not bring back memories for me (R)	0.73	0.53		
2. When listening to music I reminisce about the past	0.84	0.72		
3. When listening to music I remember my past	0.82	0.68		
4. Listening to music reminds me of people from my past	0.81	0.66		
**Anger regulation**			0.90	3.68 (0.69)
1. When I feel angry listening to music helps me look on the bright side	0.82	0.67		
2. When I feel angry listening to music helps me see things in a more positive light	0.78	0.62		
3. When I feel angry listening to music helps to take my mind off it	0.77	0.59		
4. When I feel angry listening to music distracts me from feelings of anger	0.73	0.53		
5. When I feel angry I listen to music that makes me happy	0.70	0.49		
6. When I feel angry listening to my favorite music makes me feel happier	0.73	0.54		
7. When I feel angry I get comfort from listening to music	0.71	0.51		
**Anxiety regulation**			0.90	3.82 (0.62)
1. When I feel anxious listening to music helps me look on the bright side	0.75	0.56		
2. When I feel anxious listening to music helps me see things in a more positive light	0.76	0.58		
3. When I feel anxious listening to my favorite music makes me feel happier	0.74	0.54		
4. When I feel anxious I listen to music that makes me happy	0.69	0.49		
5. Listening to music distracts me from feelings of anxiety	0.72	0.52		
6. When I feel anxious listening to music helps to take my mind off it	0.77	0.60		
7. When I feel anxious I get comfort from listening to music	0.74	0.55		
**Awe and appreciation**			0.82	4.03 (0.73)
1. Listening to music I feel a sense of awe for the talent of the composer	0.85	0.72		
2. Listening to music I feel a sense of awe for the talent of the performer	0.83	0.69		
3. When listening to music I do not admire the talent of the performers (R)	0.66	0.44		
**Loneliness regulation**			0.87	3.74 (0.80)
1. I feel less lonely when I listen to music	0.82	0.67		
2. Listening to music reduces feelings of loneliness	0.82	0.67		
3. Listening to music makes me feel less alone	0.86	0.73		
**Cognitive regulation**			0.81^r^	3.06 (1.09)
1. Playing music in the background helps me to concentrate	0.95	0.90		
2. Having background music makes it easier to focus on what I'm doing	0.86	0.74		
**Identity**			0.84	3.73 (0.82)
1. Music listening is a fundamental part of who I am	0.67	0.44		
2. The music I listen to expresses who I am as a person	0.66	0.44		
3. Listening to music has helped me discover who I am	0.82	0.67		
4. Listening to music has helped me to understand myself	0.85	0.72		

*β, regression coefficient (i.e., factor loading); r^2^, % of variance in the latent construct explained by item; α, Cronbach's alpha*;

r *, Pearson's r (provided for 2 item scales); R, reverse scored item*.

Intercorrelations between the 11 subscales (see Table 5) suggest they measure related, yet distinct, constructs. Inspection of factor scale items led to the following interpretations. Factor 1, *Stress Reduction* reflects the use of music for distraction, escape and comfort when stressed. Factor 2, *Strong Emotional Experiences* taps into intense and blended emotional experiences afforded by music listening. Factor 3, *Rumination* measures dwelling and focusing on sadness and anxiety in music listening. Factor 4 focuses on music as an aid to *Sleep*. Factor 5 represents *Reminiscence* as an expected outcome of music listening. Factors 6 and 7 measure listeners' beliefs that music provides positive reappraisal, positive emotions, distraction and comfort for *Anger Regulation* and *Anxiety Regulation*, respectively. Factor 8, measures a sense of *Awe and Appreciation* during music listening. Factor 9, *Loneliness Regulation* captures listeners' expectations that listening to music reduces feelings of loneliness. Factor 10, *Cognitive Regulation* indicates beliefs of increased concentration and focus when listening to music. Factor 11, *Identity* refers to listening to music to develop and express the self.

**Table 5:** Bivariate correlations between factors of the AFML scale in the development sample (below the main diagonal), and bivariate correlations between factors of the AFML scale in the confirmatory sample (above the main diagonal).

**AFML Factors**	**1**	**2**	**3**	**4**	**5**	**6**	***7***	**8**	**9**	**10**	**11**
1. Stress regulation	1	0.29^***^	−0.07	0.18^***^	0.18^***^	0.65^***^	0.77^***^	0.22^***^	0.49^***^	0.21^***^	0.43^***^
2. Strong emotional experiences	0.35^***^	1	0.32^***^	0.10^*^	0.51^***^	0.25^***^	0.30^***^	0.36^***^	0.27^***^	0.10^*^	0.51^***^
3. Rumination	0.14^***^	0.40^***^	1	0.05	0.21^***^	−0.02	−0.07	−0.03	0.02	0.01	0.15^***^
4. Sleep	0.25^***^	0.17^***^	0.16^***^	1	0.14^***^	0.17^***^	0.21^***^	0.12^**^	0.18^***^	0.34^***^	0.23^***^
5. Reminiscence	0.24^***^	0.43^***^	0.29^***^	0.13^**^	1	0.17^***^	0.20^***^	0.28^***^	0.19^***^	0.02	0.30^***^
6. Anger regulation	0.53^***^	0.20^***^	0.08	0.18^***^	0.16^***^	1	0.73^***^	0.17^***^	0.49^***^	0.25^***^	0.38^***^
7. Anxiety regulation	0.61^***^	0.28^***^	0.04	0.23^***^	0.16^***^	0.62^***^	1	0.25^***^	0.53^***^	0.21^***^	0.43^***^
8. Awe and admiration	0.32^***^	0.51^***^	0.16^***^	0.11^**^	0.29^***^	0.16^***^	0.24^***^	1	0.25^***^	0.12^***^	0.40^***^
9. Loneliness regulation	0.46^***^	0.35^***^	0.18^***^	0.24^***^	0.21^***^	0.42^***^	0.46^***^	0.25^***^	1	0.24^***^	0.44^***^
10. Cognitive regulation	0.26^***^	0.18^***^	0.16^***^	0.42^***^	0.10^*^	0.26^***^	0.26^***^	0.14^**^	0.23^***^	1	0.24^***^
11. Identity	0.42^***^	0.69^***^	0.32^***^	0.23^***^	0.34^***^	0.24^***^	0.35^***^	0.51^***^	0.43^***^	0.27^***^	1

* *p < 0.05*,

** *p < 0.01*,

*** *p < 0.001*.

*N = 637 (Development sample) N = 554 (Confirmatory sample)*.

### Reliability

Cronbach alpha 1951 was used to assess scale score reliability, with values of at least 0.70 indicating acceptable internal consistency (Nunnally, 1978).

#### Internal consistency

Results support the reliability of the AFML scale in both samples. Cronbach's alpha coefficients for all subscales were high suggesting good internal consistency of the measure and its subscales (see Tables 3, 4).

### Validity

#### Construct validity

Gender differences in FML have been noted with females using music for affect regulation more than males (North et al., 2000; Sloboda et al., 2001). Saarikallio (2008) found that in a sample of young people (aged 10–20 years) scores on her Music in Mood Regulation scale were significantly higher for females than males indicating greater endorsement of the affect regulation effects of music. Independent *t*-tests are expected to find, as in previous research, that scores on the affect regulation FML are significantly higher among females.

Gender differences were observed on a number of AFML factors across two samples of participants. Specifically, in the development sample females scored significantly higher than males on the following factors^1^: *Stress Regulation* [males *M* = 3.95, *SD* = 0.77, females *M* = 4.15, *SD* = 0.67; *t*_(635)_ = −3.36, *p* = 0.001], *Anger Regulation* [males *M* = 3.53, *SD* = 0.86, females *M* = 3.70, *SD* = 0.84; *t*_(635)_ = −2.36, *p* = 0.02], *Anxiety Regulation* [males *M* = 3.80, *SD* = 0.77, females *M* = 3.98, *SD* = 0.69, *t*_(635)_ = −2.96, *p* = 0.005], *Loneliness Regulation* [males *M* = 3.73, *SD* = 0.79, females *M* = 3.95, *SD* = 0.75, *t*_(635)_ = −3.39, *p* = 0.001] and *Sleep* [males *M* = 2.93, *SD* = 1.16, females *M* = 3.15, *SD* = 1.24; *t*_(635)_ = −2.22, *p* = 0.03]. Females in the confirmatory sample had significantly higher scores than males on the factors of *Anger Regulation* [males *M* = 3.52, *SD* = 0.67, females *M* = 3.75, *SD* = 0.68, *t*_(551)_ = −3.70, *p* < 0.001], *Anxiety Regulation* [males *M* = 3.69, *SD* = 0.58, females *M* = 3.87, *SD* = 0.64, *t*_(551)_ = −3.04, *p* = 0.003] and *Reminiscence* [males *M* = 3.95, *SD* = 0.79, females *M* = 4.13, *SD* = 0.69, *t* = −2.71, *p* = 0.01]. Confirming hypotheses and providing evidence of construct validity, scores on affect regulation factors were significantly higher for female relative to male respondents in both samples of participants.

#### Convergent validity

To evaluate the convergent validity of the constructs being measured, proposed relationships between subscales of the AFML scale and wellbeing outcomes were assessed by Pearson's correlations. Hypotheses were confirmed and results are presented in Table 6.

**Table 6:** Bivariate correlations between AFML factors and wellbeing measures.

	**Development sample (*N* = 637)**			**Confirmatory sample (*N* = 554)**				
**AFML factors**	**PA**	**NA**	**SWL**	**PA**	**NA**	**SWL**	**PWB**	**SocWB**
1. Stress regulation	0.03	0.04	0.00	0.08^*^	0.00	0.04	0.04	0.02
2. Strong emotional experiences	0.04	0.20^**^	−0.10^*^	0.14^**^	0.18^**^	−0.01	0.07	−0.02
3. Rumination	−0.04	0.13^**^	−0.01	−0.03	0.20^**^	−0.12^**^	−0.13^**^	−0.09^*^
4. Sleep	−0.03	−0.01	−0.07	0.04	0.09^*^	0.03	−0.01	0.05
5. Reminiscence	0.04	0.11^**^	0.00	0.08	0.10^*^	0.05	0.07	0.03
6. Anger regulation	0.15^**^	−0.02	0.11^**^	0.21^**^	−0.03	0.16^**^	0.14^**^	0.11^*^
7. Anxiety regulation	0.13^**^	−0.05	0.09^*^	0.16^**^	−0.05	0.13^**^	0.12^**^	0.09^*^
8. Awe and admiration	0.05	0.09^*^	−0.01	0.08	0.07	−0.01	0.05	0.04
9. Loneliness regulation	0.01	0.06	−0.01	0.09^*^	0.04	0.02	0.01	0.04
10. Cognitive regulation	0.09^*^	−0.05	−0.01	0.01	0.01	−0.03	−0.03	0.00
11. Identity	0.12^**^	0.13^**^	−0.07	0.05	0.05	0.07	0.11^*^	0.04

* *p < 0.05*,

** *p < 0.01*,

^***^p < 0.001. PA, Positive Affect; NA, Negative Affect; SWL, Satisfaction With Life; PWB, Psychological Wellbeing; SocWB, Social Wellbeing.

**Figure 1:**
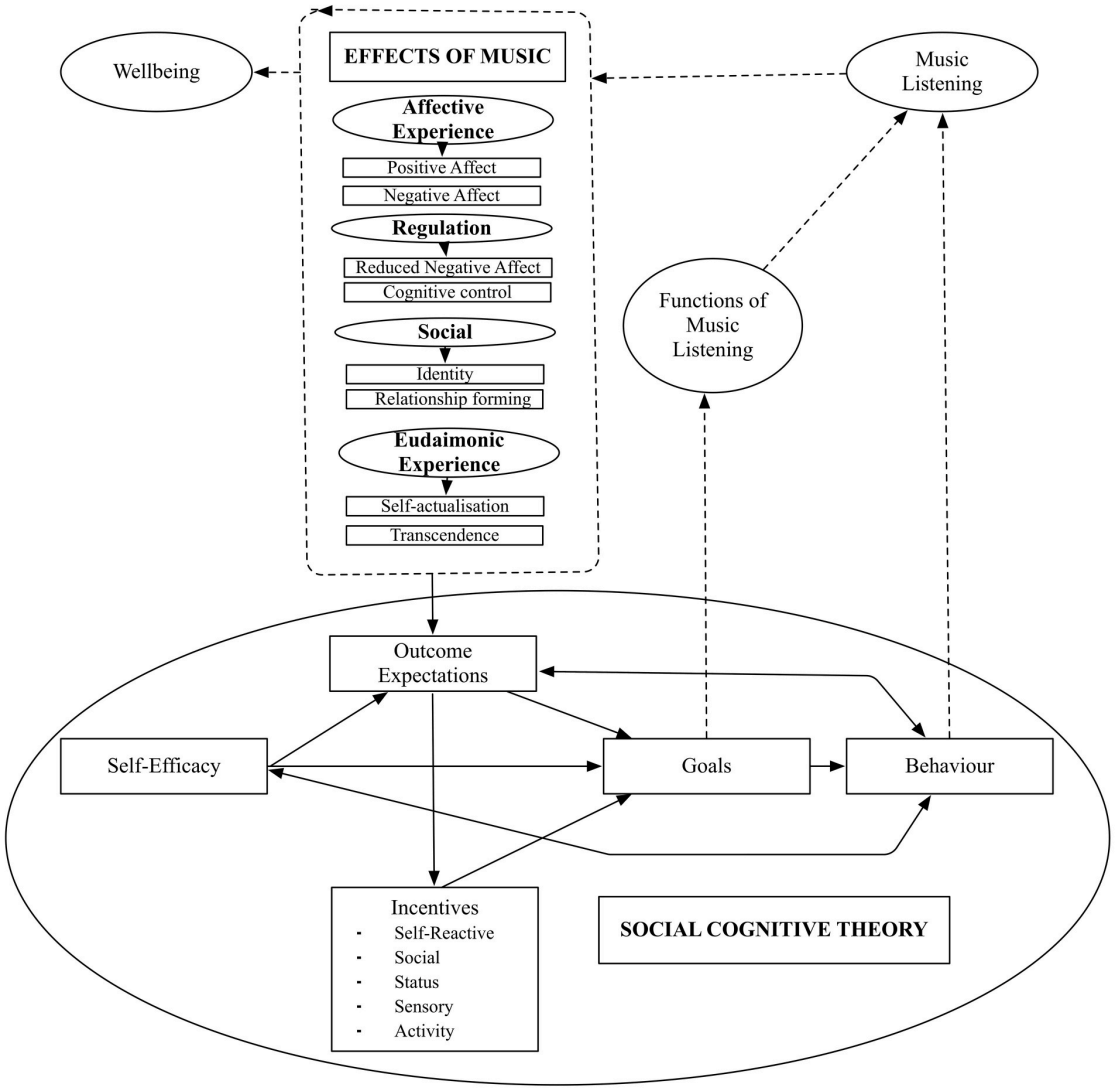
Adaptive effects of music listening identified in the literature linked with functions of music listening via Social Cognitive Theory (Bandura, 1989, 2001).

The AFML scale includes a number of affect regulation subscales: *Stress Regulation, Anger Regulation*, and *Anxiety Regulation*. To validate these subscales convergence with the emotion regulation questionnaire (ERQ; Gross and John, 2003) was examined. The ERQ measures two regulation strategies: Reappraisal and Suppression. The first strategy, reappraisal, is considered the most effective strategy in regulating NA (Augustine and Hemenover, 2009) and higher reappraisal scores as measured by the ERQ have been associated with higher SWB (Gross and John, 2003). Items measuring positive reappraisal were developed for each AFML affect regulation subscale (see Figure 1). Positive reappraisal items loaded on the factors of *Anger Regulation* and *Anxiety Regulation*. It is expected therefore that scores on these factors will converge (i.e., correlate positively) with reappraisal as measured by the ERQ. The second strategy, suppression, involves the inhibition of positive and negative emotional expression. Suppression was unrelated to music listening functions in validation tests of other FML measures, specifically, the MUSE (Chin and Rickard, 2012) and the MMR (Saarikallio, 2008). There are no items measuring the strategy of suppression in music listening on the AFML scale. Additionally, in the regulation of NA it is a largely ineffective strategy (Larsen and Prizmic, 2004). Thus suppression is not expected to correlate with affect regulation FML, demonstrating divergent validity. Further evidence of divergent validity will be provided by the lack of significant associations between scores on the emotion regulation questionnaire and scores on AFML scale factors not measuring emotion regulation (i.e., *Strong Emotional Experiences, Sleep, Reminiscence, Awe and Admiration, Cognitive Regulation*, and *Identity*). Results are presented in Table 7. Hypotheses were confirmed with two exceptions—scores on the *Rumination* factor of the AFML scale were positively associated with Suppression, and *Sleep* was associated with higher Reappraisal scores as measured by the ERQ.

**Table 7:** Bivariate correlations between the AFML subscales and the music engagement subscales of the MUSE and ERQ.

**AFML factors**	**MES**					**ERQ**	
	**I**	**II**	**III**	**IV**	**V**	**Reappraisal**	**Suppression**
1. Stress regulation	0.48^**^	0.10^*^	0.26^**^	0.25^**^	0.17^**^	0.21^**^	−0.00
2. Strong emotional experiences	0.42^**^	0.22^**^	0.27^**^	0.10^*^	0.20^**^	0.01	−0.07
3. Rumination	0.16^**^	0.10^*^	0.11^**^	0.04	0.07	−0.16^**^	0.11^**^
4. Sleep	0.20^**^	0.06	0.19^**^	0.10^*^	0.07	0.08^*^	0.04
5. Reminiscence	0.26^**^	0.11^**^	0.25^**^	0.13^**^	0.11^*^	0.05	−0.06
6. Anger regulation	0.40^**^	0.14^**^	0.24^**^	0.28^**^	0.23^**^	0.25^**^	−0.07
7. Anxiety regulation	0.44^**^	0.16^**^	0.25^**^	0.27^**^	0.21^**^	0.23^**^	−0.04
8. Awe and admiration	0.33^**^	0.25^**^	0.23^**^	0.11^*^	0.11^*^	0.07	−0.04
9. Loneliness regulation	0.42^**^	0.09^*^	0.24^**^	0.18^**^	0.15^**^	0.17^**^	0.00
10. Cognitive regulation	0.29^**^	0.02	0.17^**^	0.15^**^	0.06	0.08	0.04
11. Identity	0.53^**^	0.23^**^	0.46^**^	0.18^**^	0.24^**^	0.02	−0.07
	**MUSE**						
	**Total**	**IML**	**IMIP**	**IMT**			
AFML-Total scale (46 items) - Study 2	0.44^**^	0.38^**^	0.05	−0.01			
AFML-Total scale (48 items) - Study 1	N/A	0.45^**^	0.16^**^	0.01			

* *p < 0.05*,

** *p < 0.01*,

^***^p < 0.001; MUSE, Music Use Questionnaire; MES, Music Engagement Styles subscales; I, Cognitive and Emotional Regulation; II, Engaged Production; III, Social Connection; IV, Physical Exercise; V, Dance; ERQ, Emotion Regulation Questionnaire; MUSE IML, Index of Music Listening; IMIP, Index of Music Instrument Playing; IMT, Index of Music Training.

#### Concurrent validity

Bivariate correlations (Pearson's *r*) between the AFML subscales and another general measure of music listening functions (the MUSE, Chin and Rickard, 2012) examined the concurrent validity of the AFML scale. Results are presented in Table 7. It was not expected that the factor of *cognitive and emotional regulation* on the MUSE would correlate with all 11 subscales of the AFML scale. Reassuringly, the largest correlations were with the affect regulation subscales, however, the positive correlation with the *Identity* subscale was slightly larger. It was interesting that there was consistently positive, albeit modest, correlations between all subscales of the AFML scale and scores on the MUSE *social connection factor*, as only one social FML was retained in the current study (*Identity*). Again, the relationship between these two factors was the strongest. Such widespread associations across the factors were not predicted, however the remainder of the correlations were low and positive. This is indicative of convergence between the MUSE and the AFML scale, but also suggests there are distinctions between the constructs measured by each scale. Demonstrating concurrent validity of the AFML scale, correlations were considerably stronger between subscales of the AFML scale and the MUSE (i.e., two measures of the same construct), than between scores on the AFML scale and scores on measures of wellbeing (i.e., two measures of related constructs).

## Discussion

The AFML scale is a 46-item measure composed of 11 factors. Namely, Stress Regulation, Anxiety Regulation, Anger Regulation, Loneliness Regulation, Rumination, Reminiscence, Strong Emotional Experiences, Awe and Appreciation, Cognitive Regulation, Identity, and Sleep. In line with past research, factors relating to affective, social and cognitive functions of music listening were extracted. Contrary to the literature and our previous qualitative work, eudaimonic FML did not emerge as significant factors. The scale and its subscales possess good internal consistency and construct validity.

Informed by established guidelines in scale development (DeVellis, 2012) and in psychometric statistical analysis (Costello and Osborne, 2005; Worthington and Whittaker, 2006; Byrne, 2010), the rigorous scale development process undertaken in this large-sample study has contributed to the creation of a high quality measure of FML. In developing this measure listeners were consulted directly in a qualitative inquiry of the FML (Groarke and Hogan, 2016). These proposed constructs were expanded upon by review of the music psychology literature, and by importing theories from general and positive psychology. Padgett (1998) advocates for a mixed-methods approach to scale construction—with qualitative study to explore concepts preceding quantitative work. When initially grounded in qualitative work the psychological concepts developed in quantitative research are said to have greater validity because they are derived from real life experiences and observations (Rowan and Wulff, 2007).

Following best practice, structural equation modeling (SEM) using data from an independent sample of participants was used in the current study to confirm the factor structure identified using exploratory factor analysis (EFA) in the development sample. SEM performs best when the model being evaluated is grounded in theory (Byrne, 2010). The measurement model evaluated in the current study provided a very good fit of the data. As is always the case with SEM, there may be alternative models which fit the data equally well. In the case of scale development work alternative models could arise if alternative criteria were adopted during the EFA process. That being said, confidence in the dimensionality of the AFML measure is enhanced in that not only was the factor structure replicated in an independent sample using CFA, the measurement model evaluated was initially grounded in qualitative enquiry and further refined through a synthesis with existing theory and research, expert review, and well-established and conservative criteria for the extraction and identification of factors in EFA. The last step in particular, the exploratory factor analysis, is essential to ensure an unbiased approach to the identification of statistically reliable factors that have some chance of being confirmed in subsequent research.

Providing support for the concurrent validity of the measure, the AFML scale was moderately and positively correlated with another measure of music listening functions (i.e., the MUSE; Chin and Rickard, 2012). At the same time the broad range of factors extracted and confirmed in this study builds upon existing measures and allows for a broader investigation of the adaptive functions of music listening. Further, the AFML scale was highly correlated with the MUSE Index of Music Listening, and reassuringly, was not related to its Index of Musical Training in either sample. It was modestly correlated with an Index of Musical Instrument Playing. Subscales measuring affect regulation were also positively correlated with a standardized measure of emotion regulation from the mainstream psychology literature (i.e., the ERQ; Gross and John, 2003), in line with expectations. Positive relationships between the affect regulation factors (*Stress, Anger, Anxiety*, and *Loneliness regulation*) and the reappraisal subscale of the ERQ, as well as the lack of relationships between these same AFML factors and the suppression subscale of the ERQ was in line with predictions and provides support for the convergent and divergent validity of these AFML scale constructs. Providing further evidence of divergent validity, the affective experience factors (*Strong Emotional Experiences* and *Reminiscence*), cognitive factors (*Cognitive Regulation, Awe and Appreciation*), everyday listening factors (*Sleep*), and social factors (*Identity*) were not significantly associated with either of the emotion regulation subscales on the ERQ. These findings highlight AFML factors as discrete and meaningfully distinct functions of music listening.

Construct validity was also assessed by examining correlates between FML factors and wellbeing, specifically subjective, psychological, and social wellbeing measures. These constructs and their relationship to wellbeing outcomes are discussed below.

### Affective functions

#### Affective experience

The literature on music listening functions and the results of our qualitative work suggested people use music to generate affective experiences, such as positive and negative affect, intense emotional experiences, and also to experience reminiscence or nostalgia (Sloboda, 1999; Sloboda et al., 2001; Gabrielsson, 2002; Juslin et al., 2008; Groarke and Hogan, 2016). In the current study factors related to *Strong Emotional Experiences* and *Reminiscence* in music listening were extracted. It was predicted that higher scores on the affective experience factors would be associated with greater positive and negative affect. Notably, *Reminiscence* was associated with higher negative affect (NA) only, but *Strong Emotional Experiences* was associated with both PA and NA across samples as predicted. Affective functions including *Strong Emotional Experiences* and *Reminiscence* were among participants' highest ranked FML for enhancing wellbeing (Groarke and Hogan, 2016), but were not associated with higher psychological, and social wellbeing outcomes in the current study.

#### Affective regulation

**Figure 2:**
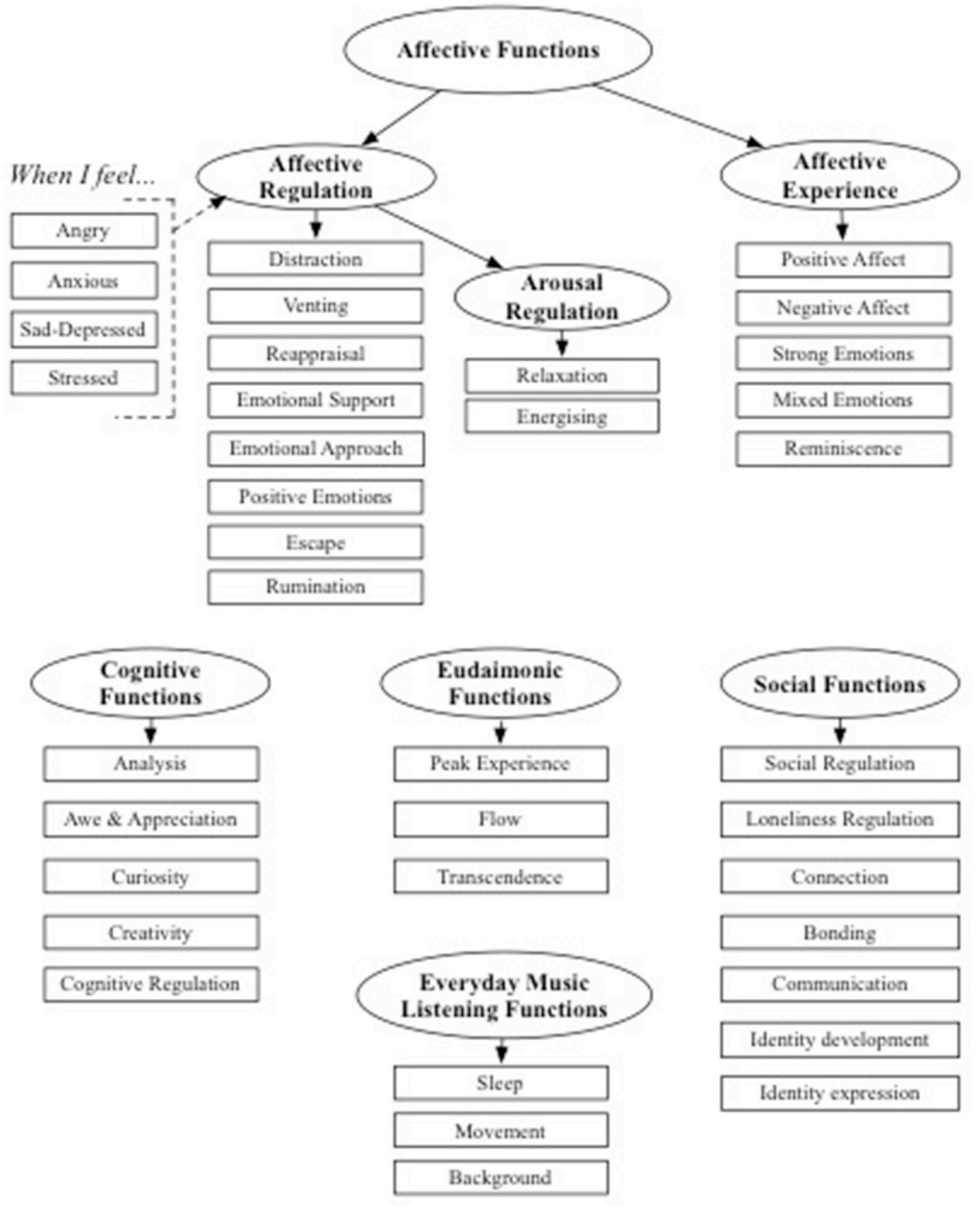
Conceptual structure informing development of the adaptive functions of music listening scale.

Supporting Juslin and Sloboda's (2010) assertion that mood regulation is the most important function of music, the majority of FML factors in the current study were affect regulatory functions. Factors emerged relating to the use of music for *Anger Regulation, Anxiety Regulation* and *Stress Regulation*. A diverse set of affect regulation strategies were derived from music psychology (Saarikallio and Erkkila, 2007) and the general psychology literature on mood regulation (Larsen, 2000) and coping (Folkman and Lazarus, 1985; Carver et al., 1989). A number of these proposed strategies (see Figure 2) were retained in factor analysis and endorsed as musical affect regulation strategies by two large samples of respondents in the current study (i.e., distraction, reappraisal, emotional support, positive emotions, escape, and rumination). As predicted, higher scores on affect regulation factors (*Anger Regulation* and *Anxiety regulation*) were significantly associated with some core indicators of enhanced SWB (i.e., higher PA and life satisfaction, but not lower NA). In the confirmatory sample, in addition to higher SWB, higher scores on these regulation factors were also associated with greater psychological and social wellbeing. In line with past research (North et al., 2000; Miranda and Claes, 2009), the use of music for affect regulation was significantly greater among females. Although *Stress Regulation* scores were also significantly higher in female respondents, the *Stress Regulation* factor was not associated with SWB as expected, with the exception of one small positive correlation with PA in the confirmatory sample. Consistent with research on music listening by Knobloch and Zillmann (2002), Saarikallio (2011), and Saarikallio et al. (2015), a factor measuring the mood regulation strategy of *Rumination* in music listening was also extracted. Items reflect music-induced rumination on sadness and anxiety. Rumination is often considered a maladaptive regulation strategy, due to its role in the maintenance of negative affective states (Aldao et al., 2010). This is also true of musical rumination (Miranda and Gaudreau, 2010). In the current study, *Rumination* was associated with lower subjective, psychological, and social wellbeing. According to theory women are more likely to use rumination (Nolen-Hoeksema, 1991), and this was also true of *Rumination* FML in the current study.

### Social functions

Listening to music for functions of identity development and expression are documented in qualitative and quantitative research with participants of all ages (Hays and Minichiello, 2005; Laukka, 2007; Lonsdale and North, 2011). The formation of identity is a cornerstone in theories of psychosocial development (Erikson, 1959). Further, development and maintenance of self-concept is an important motive driving social interaction, and is of particular significance in youth development (Carstensen, 1995). Identity has been previously linked to enhanced eudaimonic wellbeing (Bauer et al., 2006), and in the current study higher scores on the *Identity* factor was associated with higher scores on a measure of psychological wellbeing that included constructs such as *self-acceptance* and *personal growth*.

Previous research has proposed a number of social FML, such as the facilitation of social relationships (Huron, 2001; Panksepp and Bernatsky, 2002). However, few of these proposed social factors emerged in the current study. Although conceptualized as a social FML, the *Loneliness Regulation* factor was strongly related to the affect regulation subscales, and was not positively associated with social wellbeing as expected. Similar to *Stress Regulation*, however, it was modestly associated with higher PA in the confirmatory sample.

### Cognitive functions

Items pertaining to the use of music for cognitive reasons, such as regulation, music analysis, awe and appreciation, curiosity, and creativity were administered based on previous investigations and existing measures (North et al., 2000; Tarrant et al., 2000; Chamorro-Premuzic and Furnham, 2007; Chin and Rickard, 2012). A factor relating to the use of music for *Awe and Appreciation* was identified through the process of factor analysis. *Awe and appreciation* in music listening was not associated with greater psychological wellbeing as hypothesized. Unexpectedly, it was associated with greater NA in the development sample. The use of music for increased focus and concentration, *Cognitive Regulation*, also emerged as a factor in the current study, but it was not significantly associated with wellbeing as predicted, except for one small positive correlation with PA in the development sample.

A complex multi-factorial model of music listening functions was created from qualitative enquiry, and an extensive literature review. However a great many of these varied FML did not emerge as distinct factors in the context of EFA. In particular no Eudaimonic Functions were extracted. It may be that Eudaimonic FML are less distinctly identifiable among items measuring potentially more dominant Affective FML. It is also possible that Eudaimonic FML are less widely distributed in the general population, or less common in a sample of university students. Developmental differences in FML have been observed in cross-sectional surveys (Lonsdale and North, 2011), and in our qualitative work we found that Eudaimonic FML were more pronounced among older adults (Groarke and Hogan, 2016). The aim of the current study was to develop a general measure of music listening functions, and determine the latent factor structure underlying a large set of items measuring a great many hypothesized constructs. The conservative approach taken to develop a scale of high psychometric quality may have come at the expense of a more comprehensive factor structure. However, other researchers may wish to develop unidimensional scales around the wider set of constructs uncovered and can expand further upon the scale items developed and administered for EFA. To that end the full set of items are provided as Supplementary Material.

### Limitations

The large sample of participants across both studies was for the most part drawn from a convenience sample of university students. Item and page randomization of the online questionnaire was employed to minimize item-order bias (Siminski, 2008) and maximize the possibility that data lost due to attrition could be substituted later. However, despite taking these steps the high rate of attrition led to a large proportion of missing data. Although data was found to not be Missing Completely At Random (MCAR) (Little and Rubin, 1987), a complete cases analysis approach was adopted as data imputation techniques included in the SPSS software package (e.g., Expectation Maximization) are less appropriate when the proportion of missing data is greater than 5–10% (Enders, 2001; Scheffer, 2002). However, this limits the generalizability of the findings to more diverse populations.

Retention criteria recommended the removal of all but two of the negatively worded (i.e., reverse scored) items. Although it is recommended to include such items (DeVellis, 2012), some argue that negatively worded items are difficult to understand, do not reduce response bias, and should be avoided (van Sonderen et al., 2013). While potentially important from a psychometric perspective, other well-developed measures of FML have also found the inclusion of such items problematic (Saarikallio, 2008; Chin and Rickard, 2012). The factors representing Sleep and Cognitive Regulation functions of music are made up of 2 indicators each. Although factor loadings and inter-item correlations for these items were high, three to five indicators with significant loadings are recommended for factors to be sufficiently identified (Costello and Osborne, 2005). Therefore, researchers are cautioned against using these scales as unidimensional measures of Sleep and Cognitive Regulation FML.

It has been recommended that studies of music consider features of the listener, the context, and the music (Juslin and Västfjäll, 2008). The AFML scale focuses on the listener's FML, but does not fully consider the FML in context, or music preferences and choices (e.g., specific music for specific functions). At the same time, an individual's efficacy beliefs regarding the functions of music listening differ conceptually, and may in fact precede, the music selection strategies that listeners engage to fulfill these FML (North and Hargreaves, 1996, 2000; Van den Tol and Edwards, 2013). As such, the AFML scale may be used to shed light on individual differences in the listener's approach to music listening that influence how music is experienced in context. Future studies are needed to examine this issue.

The primary aim of correlation analyses in the current study was to examine the construct validity of The AFML measure. While the results may provide some insight into the nature of the relationships between adaptive FML and wellbeing outcomes, the cross-sectional nature of the research precludes causal claims being made regarding the direction of these relationships. Experimental designs provide better evidence of causation, yet the laboratory is a fairly unnatural environment for music listening. Experience and mobile sampling technologies provide considerable potential in this regard. A limitation of existing ESM studies (Sloboda et al., 2001; North et al., 2004; Juslin et al., 2008; Greasley and Lamont, 2011; Randall and Rickard, 2017) is that they have generally not included established measures of study variables; in particular none have used a psychometric measure of the FML, instead using a limited number of researcher-selected lists to track different functions, goals and reasons for listening to music in episodes of daily life. Building upon correlational studies, future research should incorporate superior predictive modeling analyses using longitudinal and experience sampling techniques in combination with high quality psychometric tools, such as The AFML Scale, to better address questions as to the relationship between adaptive FML, music listening behaviors, and wellbeing over longer periods of time in everyday contexts. At the same time, easy to administer surveys remain a valuable tool in contexts where these more complex and demanding experience sampling designs are less appropriate, for example screening in applied settings, or for use in more traditional research where longitudinal effects, or ecological validity are not the primary concern.

## Conclusion

*The Adaptive Functions of Music Listening Scale* is a measure suited for outcomes-based research on music listening functions. The AFML measure and its 11 subscales possess good internal consistency and validity. Further psychometric investigation is needed to establish the predictive, discriminant and known-groups validity of all subscales of the AFML measure. However, the affect regulation subscales in particular demonstrate good validity and reliability. Furthermore, factor scores on affective, social, and cognitive FML associated positively with indicators of wellbeing supporting the adaptivity of music listening functions.

## Ethics statement

This study was carried out in accordance with the recommendations of National University of Ireland, Galway Research Ethics Committee with written informed consent from all subjects. All subjects gave written informed consent in accordance with the Declaration of Helsinki. The protocol was approved by the National University of Ireland, Galway Research Ethics Committee.

## Author contributions

JG devised the project. JG and MH designed the study. JG collected and analyzed the data. JG and MH wrote the manuscript.

### Conflict of interest statement

The authors declare that the research was conducted in the absence of any commercial or financial relationships that could be construed as a potential conflict of interest.

## Footnotes

^1^Given multiple comparisons a more conservative alpha level of 0.005 (*p* = 0.05/11) would be more acceptable for interpreting mean differences in light of the Bonferroni approach.
