# Supplementary material for: Development and Psychometric Evaluation of the Adaptive Functions of Music Listening Scale
Source: Front Psychol. 2018 Apr 12;9:516. doi: 10.3389/fpsyg.2018.00516 (PMC5907452; doi:10.3389/fpsyg.2018.00516)
Supplement: Supplementary file 1 [file Table1.doc]

| **Table 1.** Full set of 164 scale items representing 33 hypothesised Functions of Music Listening |
| --- |
| **Affective Functions**  **Affect Regulation**  Distraction   1. Listening to music distracts me from stress 2. Listening to music distracts me from feelings of sadness/depression 3. When I feel angry, listening to music distracts me from feelings of anger 4. Listening to music distracts me from feelings of anxiety 5. When I feel stressed, listening to music helps to take my mind off it 6. When I feel sad/depressed listening to music helps to take my mind off it 7. When I feel angry, listening to music helps to take my mind off it 8. When I feel anxious, listening to music helps to take my mind off it |
| Venting   1. Listening to music helps me release feelings of stress 2. Listening to music helps me release feelings of sadness/depression 3. Listening to music helps me release feelings of anger 4. Listening to music helps me release feelings of anxiety 5. When I'm stressed, listening to music helps me release my feelings 6. When I feel sad/depressed, listening to music helps me release those my feelings 7. When I feel angry, listening to music helps me release those my feelings 8. When I feel anxious, listening to music helps me release those my feelings |
| Reappraisal   1. When I'm stressed, listening to music helps me see things in a more positive light 2. When I feel sad/depressed, listening to music helps me see things in a more positive light 3. When I feel angry, listening to music helps me see things in a more positive light 4. When I feel anxious, listening to music helps me see things in a more positive light 5. When I feel stressed, listening to music helps me look on the bright side 6. When I feel sad/depressed, listening to music helps me look on the bright side 7. When I feel angry, listening to music helps me look on the bright side 8. When I feel anxious, listening to music helps me look on the bright side |
| Emotional Support   1. When I feel stressed, I get emotional support from music 2. When I feel sad/depressed, I get emotional support from music 3. When I feel anxious, I get emotional support from music 4. When I feel angry, I get emotional support from music 5. When I feel stressed, I get comfort from listening to music 6. When I feel sad/depressed, I get comfort from listening to music 7. When I feel angry, I get comfort from listening to music 8. When I feel anxious, I get comfort from listening to music |
| Emotional Approach   1. When I'm stressed, listening to music helps me to figure out what I'm really feeling 2. When I feel sad/depressed, listening to music helps me to figure out what I'm really feeling 3. When I feel angry, listening to music helps me to figure out what I'm really feeling 4. When I feel anxious, listening to music helps me to figure out what I'm really feeling 5. When I'm stressed, listening to music helps me to delve into my feelings to get a deeper understanding of them 6. When I feel sad/depressed, listening to music helps me to delve into my feelings to get a deeper understanding of them 7. When I feel anxious, listening to music helps me to delve into my feelings to get a deeper understanding of them 8. When I feel angry, listening to music helps me to delve into my feelings to get a deeper understanding of them |
| Positive Emotions   1. When I feel stressed, listening to my favourite music makes me feel happier 2. When I feel sad/depressed, listening to my favourite music makes me feel happier 3. When I feel anxious, listening to my favourite music makes me feel happier 4. When I feel angry, listening to my favourite music makes me feel happier 5. When I feel stressed, I listen to music that makes me happy 6. When I feel sad/depressed, I listen to music that makes me happy 7. When I feel anxious, I listen to music that makes me happy 8. When I feel angry, I listen to music that makes me happy |
| Escape   1. Listening to music helps me escape from my troubles 2. I can escape stressful situations by listening to music 3. I can withdraw and escape from sad/depressing situations by listening to music 4. I can withdraw and escape from situations that make me angry by listening to music 5. I can escape situations that make me anxious by listening to music |
| Rumination   1. When I feel stressed, listening to music leads me to focus on those feelings 2. When I feel sad/depressed, listening to music leads me to focus on those feelings 3. When I feel angry, listening to music leads me to focus on those feelings 4. When I feel anxious, listening to music leads me to focus on those feelings 5. When I feel stressed, listening to music makes me dwell upon the feelings 6. When I feel sad/depressed, listening to music makes me dwell upon the feelings 7. When I feel angry, listening to music makes me dwell upon the feelings 8. When I feel anxious, listening to music makes me dwell upon the feelings   **Arousal Regulation**  Relaxation   1. Listening to music helps me to relax 2. Listening to music does not help me to relax (RS) 3. When I need to relax, I listen to music 4. When I feel tense, listening to music calms me down |
| Energising   1. When I'm tired, listening to music activates me 2. Listening to music gives me an energy boost 3. Listening to music makes me feel more alert and awake 4. Listening to music does not energise me (RS)   **Affective Experience** |
| Positive Affect   1. Listening to music gives me feelings of joy 2. Listening to music gives me feelings of elation 3. Listening to music does not gives me feelings of excitement (RS) 4. Listening to music gives me feelings of calmness 5. Listening to music gives me feelings of contentment 6. Listening to music does not gives me feelings of serenity (RS) |
| Negative Affect   1. Listening to music gives me feelings of sadness 2. Listening to music gives me feelings of melancholy 3. Listening to music does not gives me feelings of misery (RS) 4. Listening to music gives me feelings of anger 5. Listening to music gives me feelings of tension 6. Listening to music does not gives me feelings of anxiety (RS) |
| Strong Emotions   1. I feel strong emotions when listening to music 2. When listening to music, I feel intense emotions 3. I do not feel intense emotions when I listen to music (RS) 4. When listening to music, I feel emotions deeply |
| Mixed Emotions   1. When listening to music, I feel a mixture of many different emotions 2. When listening to music, I feel a range of emotions 3. When listening to music, I feel a variety of emotions simultaneously 4. When listening to music, I do not feel a mixture of emotions (RS)   Reminiscence   1. Listening to music reminds me of people from my past 2. When listening to music, I remember my past 3. Listening to music does not bring back memories for me (RS) 4. When listening to music, I reminisce about the past |
| **Cognitive Functions**  Analysis   1. I like to critically analyse a piece of music as I listen 2. I like to figure out a piece of music as I listen 3. I do not critically analyse music as I'm listening (RS) 4. Music listening is an intellectual activity pursuit for me |
| Awe & Appreciation   1. When listening to music I admire its beauty 2. When listening to music, I do not admire the talent of the performers (RS) 3. Listening to music I feel a sense of awe for the talent of the composer 4. Listening to music I feel a sense of awe for the talent of the performer |
| Curiosity   1. Discovering new music excites me my curiosity 2. Through music listening I can explore different ideas 3. Listening to music I get to experience new sensations 4. Listening to music I do not experience new ideas (RS) |
| Creativity   1. Listening to music gives me inspiration for other art forms 2. Listening to music helps me to come up with new ideas 3. I find listening to music thought provoking 4. I do not feel creative when I'm listening to music (RS) |
| Cognitive Regulation   1. Playing music in the background helps me to concentrate 2. Having music in the background does not make it easier to think (RS) 3. Having background music makes it easier to focus on what I'm doing 4. Listening to music keeps my mind occupied |
| **Eudaimonic Functions**  Peak Experience   1. I have had life changing experiences listening to music 2. I have completely lost my self in music listening 3. Listening to music I can feel a connection with something larger than myself 4. I have not had a life changing experience as a result of listening to music (RS)   Flow   1. I can lose track of time when listening to music 2. I have had moments of total absorption in music listening 3. I do not lose track of time when I'm listening to music (RS) 4. When listening to music I feel a sense of increased awareness   Transcendence   1. Listening to music opens up another world of experience 2. Listening to music does not take me to another world (RS) 3. When listening to music I am in my own private world 4. When listening to music I feel I can transcend everyday experience   **Social Functions**  Social Regulation   1. Background music helps to create a certain atmosphere when socialising 2. Background music helps people to relax more at social gatherings 3. Background music can create the mood for romance 4. Background music does not create atmosphere in social situations (RS) |
| Loneliness Regulation   1. Listening to music makes me feel less alone 2. Listening to music reduces feelings of loneliness 3. When I'm feeling lonely, music does not help (RS) 4. I feel less lonely when I listen to music |
| Connection   1. Listening to music helps me feel more connected to others 2. When I listen to music from different eras, I feel connected with the past 3. Listening to music is a way to connect with other cultures 4. When I listen to music I do not feel more connected to society (RS) |
| Bonding   1. Listening to music together is a way to bond with people 2. Shared musical experiences have helped me to bond with others 3. I have not bonded with others over shared musical experiences (RS) 4. Listening to music gives me loving feelings towards others |
| Communication   1. Listening to song lyrics is like listening to a story 2. I learn about the lives of others when listening to music 3. When listening to music, I feel like I am communicating with the song-writer 4. Music is a way to communicate with others without using language |
| Identity Development   1. Listening to music has helped me discover who I am 2. Music listening is a fundamental part of who I am 3. Listening to music has helped me to understand myself 4. My identity has not been influenced by music listening (RS) |
| Identity Expression   1. The music I listen to expresses who I am as a person 2. Through music listening I can communicate my values to others 3. Through music listening I can communicate my attitudes to others 4. Through music listening I can not communicate my self to others (RS) |
| **Everyday Music Listening Functions**  Sleep Aid   1. I listen to music in bed because it helps me get to sleep 2. Listening to music in bed helps me fall asleep 3. Listening to music does not help me fall asleep (RS) 4. I fall asleep easier when listening to music |
| Movement   1. Listening to music does not makes me feel like dancing (RS) 2. I work faster when listening to music 3. Listening to music when I'm walking helps me to keep going longer 4. Listening to music when I'm exercising helps me to keep going longer |
| Background   1. I like to have music on in the background constantly 2. Listening to music does not block out distracting background noise (RS) 3. Listening to music blocks out unpleasant background noise |

*Note;* RS = Reverse Scored item
